# Phenotypic factors associated with outcome in 977 intensive care patients with faecal peritonitis: analysis of trends in the GenOSept cohort

**DOI:** 10.1186/cc14108

**Published:** 2015-03-16

**Authors:** A Tridente, G Clarke, A Walden, A Gordon, P Hutton, J Chiche, P Holloway, G Mills, J Bion, F Stuber, C Garrard, C Hinds, GenOSept Investigators

**Affiliations:** 1 St Helens and Knowsley, Liverpool, UK

## Introduction

Patients admitted to intensive care following surgery for faecal peritonitis present particular challenges in terms of clinical management and risk assessment that require close collaboration between surgical and intensive care teams [1]. We aimed at establishing whether dynamic assessment of trends in selected variables may be associated with outcomes, and therefore inform medical decision-making.

## Methods

We analysed trends in all 35 variables available for the first week of ICU stay in 977 patients from 102 centres across 17 countries. The primary study outcome was 6-month mortality. Secondary outcomes were ICU, hospital and 28-day mortality. For each trend, Cox proportional hazards (PH) regression analyses, adjusted for age and gender, were performed for each endpoint. Trends found to be significant in these analyses, after Bonferroni correction for multiple testing, were entered into a multivariate Cox PH model, to determine independent associations with mortality.

## Results

The trends over the first 7 days of ICU stay (primary analysis) retained as independently associated with 6-month outcome were worsening thrombocytopaenia (mortality HR = 1.02, 95% CI = 1.01 to 1.03, *P *< 0.001) and changes in renal function (total daily urine output HR = 1.02, 95% CI = 1.01 to 1.03, *P *< 0.001; renal SOFA subscore HR = 0.87, 95% CI = 0.75 to 0.99, *P *= 0.047), highest recorded level of bilirubin (HR = 0.99, 95% CI = 0.99 to 0.99, *P *= 0.02) and GCS SOFA subscore (HR = 0.81, 95% CI = 0.68 to 0.98, *P *= 0.028). Changes in renal function (total daily urine output and renal component of the SOFA score), GCS component of the SOFA score, total SOFA and worsening thrombocytopaenia were also independently associated with secondary outcomes. Dynamic trends over the first 7 days of ICU stay in all other measured laboratory variables, physiological parameters or radiological findings failed to be retained as independently associated with outcome on multivariate analyses. Furthermore, changes in respiratory support, renal replacement therapy and inotropic and/or vasopressor requirements appeared not to be independently associated with any of the primary or secondary outcomes. Secondary *post hoc *analyses on trends over the first 3 and 5 days corroborated these findings.

## Conclusion

Only deterioration in renal function, thrombocytopaenia and hyperbilirubinaemia over the first 7 days of ICU stay were consistently associated with mortality at all endpoints.
